# Optimizing nitrogen and sulfur supplementation for enhanced growth and biochemical composition in *Solanum lycopersicum* under hydroponic conditions

**DOI:** 10.1038/s41598-025-26760-0

**Published:** 2026-01-18

**Authors:** Mostofa Asif Anjum, Md. Eram Hosen, Farzana Sayed Sraboni, Nazim Uddin Ahmed, Ayan Goshwami, Md. Asadul Islam, Uzzal Kumar Acharjee, Taha Alqahtani, Emad Rashad Sindi, Zsolt Tóth, Rashed Zaman, Magdi E. A. Zaki

**Affiliations:** 1 Professor Joarder DNA and Chromosome Research Laboratory, Department of Genetic Engineering and Biotechnology, University of Rajshahi, Rajshahi, 6205 Bangladesh; 2 Biomedical Science and Molecular Biology, College of Medicine and Dentistry, James Cook University, Townsville, QLD 4811 Australia; 3 Microbiology Laboratory, Department of Genetic Engineering and Biotechnology, University of Rajshahi, Rajshahi, 6205 Bangladesh; 4 Office: Drugs and Toxins Research Division, BCSIR Laboratories, Rajshahi, 6205 Bangladesh; 5 Office: BCSIR Laboratories, Rajshahi, 6205 Bangladesh; 6 Department of Pharmacology, College of Pharmacy, King Khalid University, 62529 Abha, Kingdom of Saudi Arabia; 7 Division of Clinical Biochemistry, Department of Basic Medical Sciences, College of Medicine, University of Jeddah, 23890 Jeddah, Kingdom of Saudi Arabia; 8 Faculty of Wood Engineering and Creative Industries, University of Sopron, Sopron, Hungary; 9 Department of Chemistry, College of Science, Imam Mohammad Ibn Saud Islamic University (IMSIU), 11623 Riyadh, Kingdom of Saudi Arabia

**Keywords:** Tomato, Hydroponics, Nitrogen, Sulfur, Oxidative stress, Nutrient use efficiency, Biochemistry, Biotechnology, Drug discovery, Molecular biology

## Abstract

*Solanum lycopersicum* (tomato) is a globally significant crop valued for its nutritional and economic importance. This study investigated the effects of nitrogen (N) and sulfur (S) supplementation on tomato growth, biochemical profiles, and stress responses in a hydroponic system. Five treatments were tested: T1 (0% N, 0% S), T2 (50% N, 50% S), T3 (100% N, 100% S, control), T4 (250% N, 250% S), and T5 (500% N, 500% S). Results suggest that T3 (100% N, 100% S) supported optimal growth (plant height: 35.3 cm, leaf number: 33.3, root weight: 1.48 g), while T1 exhibited severe deficiency symptoms and T5 showed signs of nutrient toxicity. Biochemical analyses revealed strong positive correlations between N/S availability and chlorophyll (*r* = 0.92, *p* < 0.01), carotenoids (*r* = 0.89, *p* < 0.01), and protein content (*r* = 0.95, *p* < 0.01). T5 displayed elevated antioxidant enzyme activities (CAT: 0.42 μmol/min/mg protein; APX: 2.40 μmol/min/mg protein) and increased cell death (40.0%), indicating metabolic stress. These findings suggest that the 100% N/S treatment was optimal for tomato growth and biochemical composition in hydroponic systems, while excessive nutrients induce stress. This study highlights the importance of precise nutrient management for sustainable hydroponic tomato production. Limitations include the use of a single cultivar, a small sample size (n = 3), and a controlled hydroponic environment, which may not fully represent field conditions.

## Introduction

Tomato (*Solanum lycopersicum L*.) is among the most globally cultivated horticultural crops, with a production volume surpassing 180 million metric tons annually^1^. Its widespread appeal stems not only from its culinary versatility but also from its substantial nutritional profile, which includes essential vitamins A, C, and K, as well as the potent antioxidant lycopene^2,3^.These compounds contribute to improved human health, reducing the risk of chronic illnesses such as cardiovascular disease and certain cancers. Tomatoes also hold considerable economic importance in both fresh and processed forms, making them a staple in food security strategies.

With escalating global challenges such as rapid urbanization, land degradation, and climate variability, the need for innovative and resource-efficient agricultural methods has intensified. Hydroponic cultivation, which involves growing plants in nutrient-rich water without soil, is now being widely adopted as a sustainable solution. This technique enables year-round production with minimal land and water inputs, often yielding 3–10 times more than conventional soil-based systems while consuming up to 90% less water^4,5^.

Nutrient management is a pivotal component in optimizing hydroponic cultivation, where every mineral must be precisely supplied. Among these, nitrogen (N) and sulfur (S) are vital macronutrients that directly impact plant physiology, morphology, and metabolic function. Nitrogen is indispensable for the biosynthesis of amino acids, nucleotides, and chlorophyll, and it plays a central role in photosynthesis and biomass accumulation^6^. In parallel, sulfur, though needed in smaller amounts, is crucial for the synthesis of sulfur-containing amino acids like cysteine and methionine, as well as important antioxidants such as glutathione^7,8^.

Although the individual roles of nitrogen and sulfur in plant development are well established, their combined and interactive effects, particularly in hydroponically grown tomatoes, remain largely unexplored. Studies suggest that sulfur availability modulates nitrogen assimilation, indicating a synergistic relationship^9,10^. Conversely, an imbalance between these nutrients can trigger metabolic stress, impairing nutrient use efficiency, altering enzymatic activities, and compromising overall plant growth. Yet, most research conducted to date has addressed nitrogen and sulfur independently, leading to a critical gap in our understanding of their combined influence on plant health in controlled environments.

This study aims to address three specific research gaps, including determining the optimal N:S ratio to support healthy tomato growth under hydroponic conditions; identifying the threshold concentrations for nutrient deficiency and toxicity; and characterizing the biochemical, morphological, and physiological responses of tomato plants exposed to varying N and S levels. Bridging this knowledge gap is vital for formulating precise nutrient solutions that enhance crop performance, resource use efficiency, and stress resilience in hydroponic systems.

By systematically evaluating the influence of N and S ratios on tomato plant development, this study provides actionable insights for improving yield and quality in soilless cultivation. The findings are expected to contribute to more sustainable, efficient, and nutritionally rich hydroponic tomato production practices, supporting the global push toward climate-resilient agriculture.

## Methods

### Plant material and growth conditions

Tomato seeds (*Solanum lycopersicum L.,* local cultivar) were collected from Nowdapara, Rajshahi, Bangladesh, surface-sterilized with 2% NaOCl, and germinated on moist filter paper at 25 °C. Seedlings were transferred to 500 mL hydroponic containers under controlled conditions: temperature 25 ± 1 °C, humidity 60–70%, photoperiod 16 h light/8 h dark, light intensity 15,000 lx, and nutrient solution pH 5.8 ± 0.2, monitored daily^11^.

### Experimental design

Five treatments (n = 3 plants per treatment) were applied with varying N/S concentrations, using NH₄NO₃ as the nitrogen source and MgSO₄ as the sulfur source, as detailed in Table 1^11^:

**Table 1:** Treatment application.

Treatment	NH₄NO₃ (mg/L)	MgSO₄ (mg/L)	N (% of Control)	S (% of Control)
T1	0	0	0	0
T2	40	90	50	50
T3	80	180	100	100
T4	200	450	250	250
T5	400	900	500	500

### Data collection

#### Morphological parameters

Plant height (cm), plant weight (g), root length (cm), root weight (g), leaf length (cm), leaf width (cm), leaf number, and leaf weight (mg) were measured after 30 days of growth using standard rulers and precision balances.

#### Biochemical assays

Several biochemical tests have been conducted, such as chlorophyll a/b, total chlorophyll, and carotenoids^12^, determination of nitrogen uptake and protein (%) in plants using the Kjeldahl method, estimation of total soluble sugar, estimation of cell death analysis, estimation of total amino acid contents, and analysis of antioxidant enzymes (CAT, APX).

Chlorophyll a, chlorophyll b, total chlorophyll, and carotenoids were quantified using the Arnon (1949) method. Fresh leaves (125 mg) were extracted in 5 mL of 80% acetone, centrifuged at 3000 rpm for 10 min, and diluted with 12.5 mL of 80% acetone. Absorbance was measured at 480, 510, 663, and 645 nm using a UV–Vis spectrophotometer (Analytic Gena, Germany)^13^.

Nitrogen concentration in plant samples was ascertained using the Kjeldahl method, which involved titration, distillation, and digestion. To transform organic nitrogen into ammonium sulfate ((NH₄)₂SO₄), the dried, ground sample was digested using concentrated sulfuric acid (H₂SO₄) and a catalyst mixture (K₂SO₄ and CuSO₄) until a clear solution was formed. After the digest was diluted and distilled using sodium hydroxide (NaOH), ammonia gas (NH₃) was released and absorbed in a solution of boric acid (H₃BO₃). Standardized acid (HCl or H₂SO₄) was used to titrate the trapped ammonia until the indicator’s color changed. The amount of acid used in the titration process was used to determine the nitrogen content.

First, a known amount of dried and finely ground plant sample is weighed and placed into a Kjeldahl digestion flask. Concentrated sulfuric acid (H₂SO₄) is added to the flask along with a catalyst mixture, such as potassium sulfate (K₂SO₄) and copper(II) sulfate (CuSO₄), to speed up the digestion process. The mixture is heated until the solution becomes clear and colorless, indicating that the organic nitrogen in the sample has been converted into ammonium sulfate ((NH₄)₂SO₄). After cooling, the digest is diluted with distilled water and transferred to a volumetric flask for further analysis^14^.

To determine the total soluble sugar concentration in the roots and leaves, the method was followed from the paper^15^ with some modification. The anthrone reagent method was used to measure the total amount of soluble sugars in plant samples^16^. After homogenizing fresh root and leaves samples (0.25 g) in 80% aqueous ethanol for 5 min at 12,000 rpm, the supernatant was gathered. After combining the extract with 0.2% anthrone reagent, it was incubated for ten minutes in a boiling water bath before being chilled on ice. A Thermo Scientific Genesys UV–Visible spectrophotometer was used to detect the optical density (OD) at 620 nm, and a standard glucose curve was used to calculate the concentration of total soluble sugar.^17^.

In plants, cell death is an essential biological process that affects defense, development, and stress reactions. With a few adjustments, the Evans blue method was applied to examine cell death in the roots and shoots following the work of^18^. The separated 0.25 g weighted root and shoot samples were first submerged for 15 min at room temperature in a 0.25% Evans blue solution. After that, 1.0 mL of 80% ethyl alcohol was added to the solution, and it was incubated for 10 min. After 15 min at 50 °C in a water bath, the samples were centrifuged for 10 min at 12,000 rpm. Next, the absorbance (Analytic Gena, Germany) was measured at 600 nm, and the fresh weight was used to compute the amount of cell death^19^.

The total amino acid contents of plant leaves were determined by using the ninhydrin colorimetric method modified by^20^. Briefly, 200 mg of leaf samples were extracted with 1 ml 200 mM KPO_4_—(_P_H 7.0) and centrifuged at 12,000 g for 10 min at 4° C, and the supernatant and mixed with ninhydrin solution. Thereafter, the mixture was incubated for 10 min, and then it was cooled on ice. Fifty percent of ethanol was added and mixed, then the absorbance of the reaction mixture was recorded at 570 nm. The total amino acid contents of plant leaves were determined by using the ninhydrin colorimetric method modified by Magné and Larher (1992)^20^. Briefly, 200 mg of leaf samples were extracted with 1 ml 200 mM KPO_4_—(_P_H 7.0) and centrifuged at 12,000 g for 10 min at 4° C, and the supernatant and mixed with ninhydrin solution. Thereafter, the mixture was incubated for 10 min, and then it was cooled on ice. Fifty percent of ethanol was added and mixed, then the absorbance of the reaction mixture was recorded at 570 nm.

Verma and Dubey (2003) were used to measure CAT and APX activities. 0.3 g samples of leaves and roots were crushed independently in phosphate buffer (10 mL, 100 mM, and pH 7.0) to test for all antioxidant activities^21^. Then, the supernatant was separated from the homogenate samples by centrifuging them for 12 min at 12,000 rpm. H_2_O_2_ (400 μl of 6%), 100 μl of leaf extract, and potassium phosphate buffer (1.5 ml, 100 mM, pH 7.0) were added to 2 mL of the reaction mixtures for CAT activity. Then, a UV–Vis spectrophotometer (Analytic Gena, Germany) was used to record the absorbance drop at 240 nm. EDTA (100 µl, 0.1 mM), epinephrine (500 µl, 0.6 mM), and sodium bicarbonate or carbonate buffer (1.3 mL, 50 mM, pH 9.8) were combined to create the reaction components for SOD. At 475 nm, the production of adrenochrome was detected (Analytic Gena, Germany). For APX, reaction mixtures were made with potassium phosphate buffer (1 mL of 100 mM, pH 7.0), ascorbic acid (500 μl of 0.2 mM), EDTA (100 μl of 0.2 mM), H_2_O_2_ (300 μl of 6%), and 100 μl of plant extract. A UV–VIS spectrophotometer (Analytic Gena, Germany) was used to measure the decrease in absorbance at 290 nm at 10-s intervals for up to one minute.

### Statistical analysis

Data were analyzed using R 4.2.1 with one-way ANOVA and Tukey’s HSD post-hoc test (α = 0.05) to assess treatment effects. Pearson correlation analysis was conducted to evaluate relationships between N/S levels and biochemical parameters, with significance set at *p* < 0.01.

## Results

### Seed germination test

**Fig. 1:**
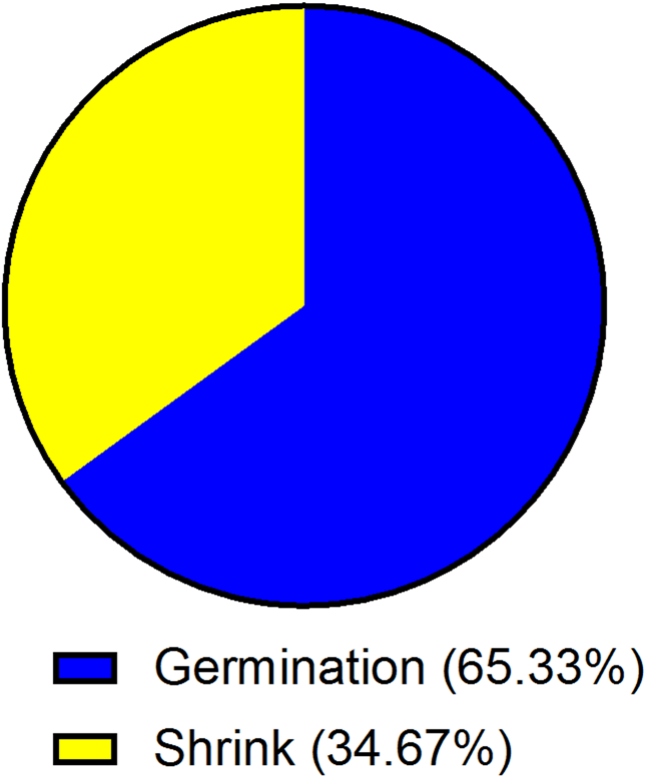
Seed germination test.

A germination test was conducted using 500 seeds distributed across five Petri dishes (100 seeds per dish). An average of 326 seeds germinated, showing normal root and shoot development, yielding a germination rate of 65.3%. Non-viable or shriveled seeds (174) indicated potential issues with seed quality or storage (Fig. 1).

### Morphological traits

#### Plant height and weight

**Fig. 2:**
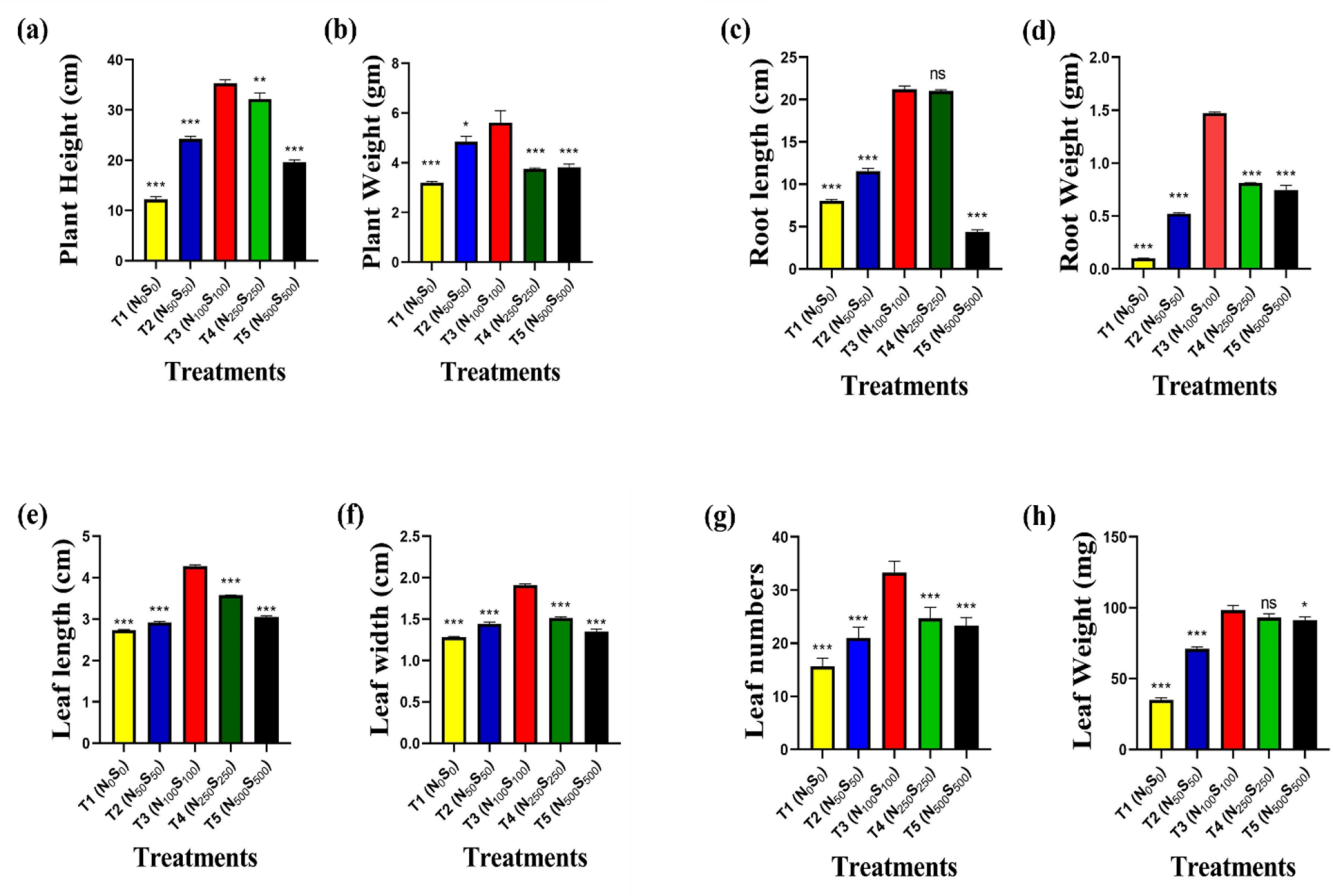
Morphological traits of *Solanum lycopersicum* under different nutrient treatments. (**a**) Plant height, (**b**) weight, (**c**) root length, (**d**) root weight, (**e**) leaf length, (**f**) leaf width, (**g**) total leaf number, and (**h**) total leaf fresh weight were measured across five treatment groups (T1–T5) differing in nutrient supply. Data are presented as mean ± SEM (n = 3 per group). Statistical significance was determined using one-way ANOVA followed by post hoc testing. *p < 0.05, **p < 0.01, ***p < 0.001.

Significant differences in plant height were observed among the five treatment groups (Fig. 2a). Plants grown under T3 (control, 100% N/S) reached the greatest height (35.3 ± 0.4 cm), significantly exceeding those under T1 (12.3 ± 0.3 cm, ***p < 0.001), T2 (24.2 ± 0.3 cm, ***p < 0.001), T4 (32.1 ± 0.7 cm, **p < 0.01), and T5 (19.6 ± 0.1 cm, ***p < 0.001). A similar trend was observed for plant biomass (Fig. 2b), with T3 exhibiting the highest fresh weight (5.6 ± 0.3 g), significantly greater than T1 (3.2 ± 0.03 g, ***p < 0.001), T2 (4.8 ± 0.1 g, *p < 0.05), T4 (3.8 ± 0.02 g, ***p < 0.001), and T5 (3.8 ± 0.08 g, ***p < 0.001).

#### Root development

Root length varied substantially among treatments (Fig. 2c). The longest roots were observed in T3 (21.2 ± 0.2 cm), comparable to T4 (21.0 ± 0.1 cm; ns), but significantly longer than roots in T1 (8.0 ± 0.1 cm; ***p < 0.001), T2 (11.6 ± 0.2 cm; ***p < 0.001), and T5 (4.4 ± 0.1 cm; ***p < 0.001). Root biomass followed the same trend (Fig. 2d), with T3 plants producing the heaviest roots (1.48 ± 0.004 g), significantly greater than T1 (0.10 ± 0.001 g), T2 (0.52 ± 0.004 g), T4 (0.81 ± 0.001 g), and T5 (0.75 ± 0.03 g) (***p < 0.001 for all comparisons).

#### Leaf morphometrics

Leaf dimensions were strongly influenced by treatment. Maximum leaf length was recorded in T3 (4.3 ± 0.02 cm), significantly longer than those in T1 (2.7 ± 0.01 cm), T2 (2.9 ± 0.02 cm), T4 (3.6 ± 0.002 cm), and T5 (3.1 ± 0.02 cm) (***p < 0.001 for all; Fig. 2e). Leaf width exhibited a parallel pattern (Fig. 2f), with T3 (1.9 ± 0.01 cm) outperforming T1 (1.3 ± 0.004 cm), T2 (1.4 ± 0.01 cm), T4 (1.5 ± 0.005 cm), and T5 (1.3 ± 0.02 cm) (***p < 0.001).

#### Leaf number and mass

A marked variation in leaf number was observed across the five treatments, with plants grown under T3 producing the highest number of leaves (33.3 ± 1.2), indicating superior vegetative growth (Fig. 2g). This value was significantly greater (*p* < 0.001) compared to all other treatments: T1 had the fewest leaves (15.7 ± 0.9), followed by T2 (21.0 ± 1.2), T5 (23.3 ± 0.9), and T4 (24.7 ± 1.2). Leaf biomass followed a similar trend (Fig. 2h). Plants in T3 exhibited the greatest mean leaf weight (98.3 ± 1.9 mg), significantly surpassing that of T1 (35.0 ± 0.9 mg, *p* < 0.001) and T2 (71.2 ± 0.8 mg, *p* < 0.001). Additionally, results also indicate that T5 (91.0 ± 1.4 mg, *p* < 0.05) caused a slight but significant reduction in leaf weight compared to the control. Although T4 (93.0 ± 1.6 mg) showed a high leaf weight comparable to T3, the difference was not statistically significant.

### Biochemical characteristics of *Solanum lycopersicum*

#### Nitrogen content

**Fig. 3:**
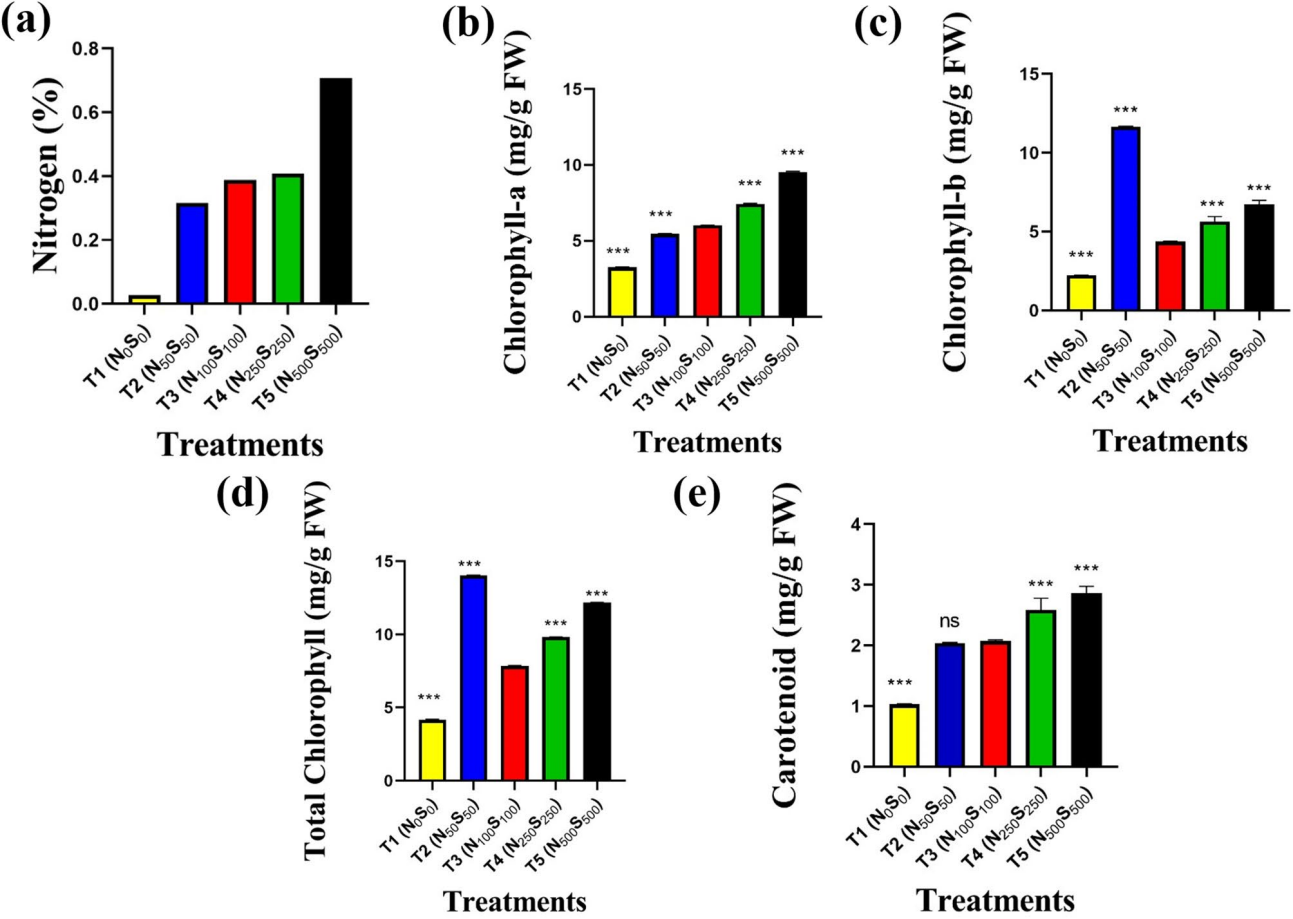
Biochemical characteristics of *Solanum lycopersicum* under different nutrient treatments. (**a**) total nitrogen content (%) measured by Kjeldahl analysis, and photosynthetic pigment concentrations: (**b**) chlorophyll-a, (**c**) chlorophyll-b, (**d**) total chlorophyll (a + b), and (**e**) carotenoids, all expressed in mg/g fresh weight (FW). Values represent mean ± SEM (n = 3 per treatment). Statistical significance was assessed using one-way ANOVA followed by post hoc tests. *p < 0.05, **p < 0.01, ***p < 0.001.

Kjeldahl analysis revealed pronounced differences in nitrogen percentage across treatments (Fig. 3a). The lowest nitrogen content was observed in T1 (0.03%), while the highest was in T5 (0.71%). Intermediate values were found in T2 (0.32%), T3 (0.39%), and T4 (0.41%), suggesting a dose-dependent response to nutrient supplementation.

#### Chlorophyll and carotenoid pigments

Photosynthetic pigment concentrations varied significantly across the different nutrient treatments. Chlorophyll-a content (Fig. 3b), measured in milligrams per gram of fresh weight (mg/g FW), was lowest in nutrient-deficient plants (T1; 0.65 ± 0.004 mg/g FW), and progressively increased across T2 (1.09 ± 0.003 mg/g FW), T3 (1.21 ± 0.002 mg/g FW), and T4 (1.49 ± 0.004 mg/g FW), reaching the highest level in T5 (1.91 ± 0.004 mg/g FW), with all comparisons showing strong statistical significance (***p < 0.001). Interestingly, the trend for chlorophyll-b content (Fig. 3c) differed. The highest concentration was observed in T2 (2.33 ± 0.003 mg/g FW), followed by T5 (1.35 ± 0.03 mg/g FW), T4 (1.13 ± 0.04 mg/g FW), and T3 (0.87 ± 0.003 mg/g FW), while T1 exhibited the lowest level (0.45 ± 0.002 mg/g FW; ***p < 0.001).

Total chlorophyll content (Fig. 3d), calculated as the sum of chlorophyll-a and chlorophyll-b, was also highest in T2 (2.81 ± 0.002 mg/g FW), followed by T5 (2.44 ± 0.001 mg/g FW), T4 (1.96 ± 0.001 mg/g FW), and T3 (1.57 ± 0.004 mg/g FW), with T1 again recording the lowest total chlorophyll level (0.83 ± 0.004 mg/g FW; ***p < 0.001). Carotenoid content (Fig. 3e) exhibited a consistent upward trend with increasing nutrient availability. T1 plants had the lowest carotenoid concentration (0.21 ± 0.001 mg/g FW), which increased across T2 (0.41 ± 0.001 mg/g FW), T3 (0.41 ± 0.002 mg/g FW), and T4 (0.52 ± 0.02 mg/g FW), peaking in T5 (0.57 ± 0.01 mg/g FW). These increases were statistically significant (***p < 0.001), indicating a clear influence of nutrient concentration on pigment biosynthesis.

#### Protein, amino acid, and sugar content

**Fig. 4:**
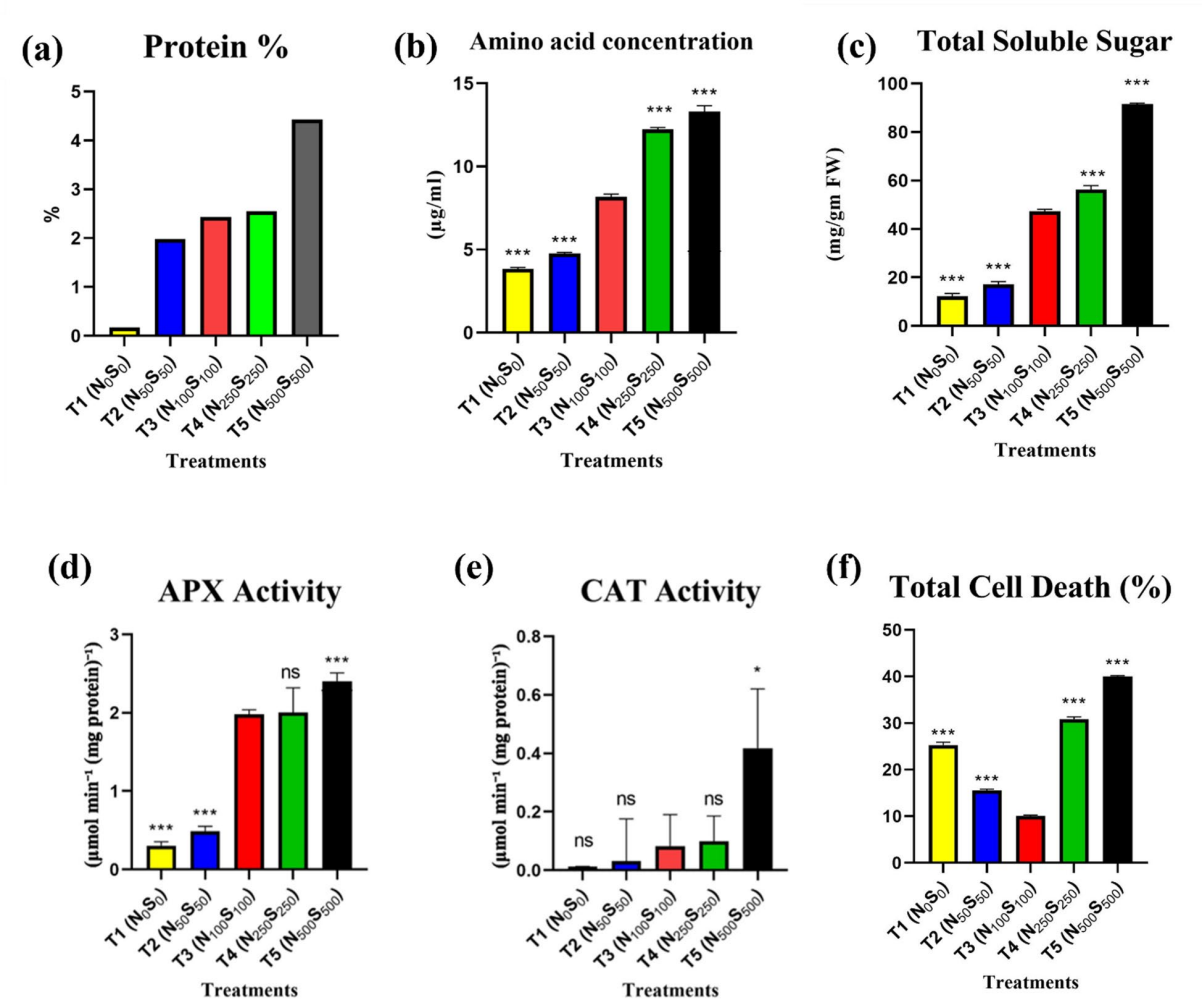
Biochemical and stress-response indicators in *Solanum lycopersicum* under different treatments. (**a**) Soluble protein content, (**b**) amino acid concentration (µg/mL), (**c**) total soluble sugar (TSS) content (mg/g FW), (**d**) APX activity, (**e**) CAT activity, and (**f**) total cell death. All values are expressed as Mean ± SEM (n = 3). Statistical significance compared to T3 is denoted as *p < 0.05; **p < 0.01; ***p < 0.001.

The biochemical composition of *Solanum lycopersicum* varied significantly across treatments. Soluble protein content (Fig. 4a) was lowest in T1 (0.17%), reflecting nutrient deficiency, but increased progressively with treatment intensity: T2 (1.98%), T3 (2.43%), and T4 (2.55%), reaching a peak in T5 (4.43%). A comparable trend was observed for free amino acid concentrations (Fig. 4b), which rose from 3.8 ± 0.05 µg/mL in T1 to 6.1 ± 0.1 in T2, 8.2 ± 0.1 in T3, and 12.2 ± 0.7 in T4, with the highest value in T5 (13.3 ± 0.2 µg/mL), indicating enhanced protein metabolism under improved nutrient conditions. Total soluble sugar (TSS) content (Fig. 4c), expressed in mg/g fresh weight (FW), showed a pronounced increase in T5 (91.5 ± 0.3), suggesting enhanced carbohydrate accumulation. This was followed by T4 (56.3 ± 0.9), T3 (47.3 ± 0.6), T2 (17.0 ± 0.8), and the lowest in T1 (12.3 ± 0.6; ***p < 0.001), indicating that nutrient-rich conditions favored sugar biosynthesis and storage.

#### Antioxidant enzyme activities

APX and CAT activities were markedly influenced by nutrient levels (Fig. 4d & e). APX activity was lowest in T1 (0.30 ± 0.03 μmol/min/mg protein), with progressive increases across T2 (0.49 ± 0.04), T3 (1.98 ± 0.04), and T4 (2.01 ± 0.18), reaching a peak in T5 (2.40 ± 0.06; ***p < 0.001). A similar pattern was seen for CAT activity, ranging from 0.01 ± 0.0004 in T1 to 0.42 ± 0.12 in T5 (*p < 0.05), with intermediate levels in T2, T3, and T4.

#### Cell death percentage

Despite enhanced biochemical performance, excessive nutrient treatment T5 (40.0 ± 0.1%) and T4 (30.8 ± 0.3%, ***p < 0.001) led to the highest cell death percentages (indicating severe stress/toxicity), whereas T3 (optimal N/S) (10.1 ± 0.1%) had the lowest cell death (healthiest outcome) (Fig. 4f).

#### Pearson correlation analysis

Pearson correlation analysis revealed strong positive correlations between N/S availability and key biochemical parameters: chlorophyll (*r* = 0.92, *p* < 0.01), carotenoids (*r* = 0.89, *p* < 0.01), and protein content (*r* = 0.95, *p* < 0.01), confirming the influence of nutrient levels on metabolic responses.

## Discussion

Nitrogen and sulfur are closely linked in plant metabolism, particularly in amino acid synthesis, protein formation, and chlorophyll production^6^. Nitrogen is a primary component of amino acids, proteins, chlorophyll, and nucleic acids, making it crucial for plant growth, photosynthesis, and overall development. Sulfur is a vital element in the synthesis of amino acids (e.g., cysteine and methionine), vitamins, and coenzymes, and it plays a key role in nitrogen metabolism and chlorophyll production^22^. Nitrogen and sulfur interact closely in plant metabolism. Sulfur is required for the efficient use of nitrogen, and a sulfur deficiency can limit nitrogen utilization, even if nitrogen is abundant^23^.

Nitrogen deficiency causes stunted growth, yellowing of older leaves (chlorosis), and reduced protein synthesis. Sulfur deficiency causes yellowing of younger leaves (similar to nitrogen deficiency but starts in younger leaves), reduced growth, and poor protein synthesis^24^. Sulfur-containing compounds, such as glutathione and phytochelatins, play a critical role in plant defense mechanisms against oxidative stress and heavy metal toxicity. Sulfur-containing compounds, such as glutathione and phytochelatins, play a critical role in plant defense mechanisms against oxidative stress and heavy metal toxicity^25^. Balanced application of nitrogen and sulfur fertilizers is critical for optimizing crop yield and quality. Excessive nitrogen without sufficient sulfur can lead to inefficient nitrogen use and environmental pollution^26^.

Proper management of nitrogen and sulfur fertilization can reduce environmental impacts, such as nitrate leaching and greenhouse gas emissions, while improving crop resilience and nutritional quality^27^. Nitrogen and sulfur are indispensable for plant growth, playing critical roles in protein synthesis, chlorophyll production, and stress tolerance. Their synergistic relationship highlights the importance of balanced fertilization to optimize crop yield, quality, and environmental sustainability. Proper management of these nutrients is essential for sustainable agriculture.

Tomato seed germination is influenced by several factors, including temperature, moisture, light, and seed quality. Optimal germination occurs at temperatures between 20 and 30 °C, with consistent moisture but not waterlogging. Pre-treatment methods such as seed soaking or priming can enhance germination rates. Proper seed storage and handling are also critical to maintaining seed viability.^28,29^. The germination rate of tomato seeds typically ranges between 70 and 90% under optimal conditions. Factors such as seed quality, temperature, moisture, and soil conditions can influence this rate. High-quality seeds stored properly can achieve germination rates closer to 90%, while older or improperly stored seeds may see rates drop to 70% or lower. In our experiment, we found that the germination rate was 65.33% **(**Fig. 1**).**

These macronutrients further affect plant morphology, and adequate availability of these nutrients promotes healthy growth and development, while deficiencies or imbalances can lead to stunted growth and reduced biomass production. Nitrogen primarily enhances vegetative growth, including plant height, leaf number, and biomass, while sulfur plays a critical role in chlorophyll synthesis, protein formation, and root development. Both nutrients interact synergistically to optimize plant morphology^25,30^. Nitrogen increases plant height by promoting cell division and elongation. Sulfur supports stem strength and elongation, but its effect is less pronounced compared to nitrogen^31^. In our study, we also found that increasing concentration up to the optimum level helps to increase the morphological traits and then overdosing stunts growth **(**Fig. 2a & b**).**

Significantly increases shoot and root biomass due to its role in protein synthesis and photosynthesis. Sulfur enhances biomass by improving nutrient uptake and metabolic processes. Leaf Number nitrogen stimulates leaf initiation and expansion, leading to a higher number of leaves. Sulfur ensures proper leaf development and prevents premature leaf senescence^25^. Our experiment supported this data **(**Fig. 2e & f**).** In the case of leaf length and width, Nitrogen promotes larger leaf size by enhancing cell expansion and chlorophyll content. Sulfur maintains leaf structure and prevents chlorosis, ensuring optimal leaf dimensions^32^. In our experiment, we also found that increasing concentration up to the optimum level helps to increase the morphological traits and then overdosing stunts growth **(**Fig. 2**)**. Our results show that T3 (N_100_S_100_) provided the best conditions for plant growth, with significantly higher plant height, leaf numbers, and biomass. These findings align with previous studies, indicating that optimal N and S availability enhances photosynthetic efficiency and biomass accumulation^33^. Conversely, T1 (N_0_S_0_) showed severe growth reduction, evident from the lowest root and shoot biomass. Nitrogen deficiency is known to cause chlorosis due to reduced chlorophyll content, while sulfur deficiency impairs protein synthesis^25^. This was supported by the lowest levels of chlorophyll and protein recorded in T1.

In the case of Root Length, Nitrogen encourages root growth, but excessive nitrogen can shift allocation to shoots at the expense of roots. Sulfur improves root elongation and branching, enhancing nutrient and water uptake. Both nutrients are vital for chlorophyll synthesis, enzyme activity, and stress tolerance. A deficiency in either nutrient can lead to reduced growth, yellowing of leaves (chlorosis), and poor yield. Plants treated with higher nitrogen concentrations will generally uptake more nitrogen, and this increase can be detected using the Kjeldahl method. However, the method measures total nitrogen and does not distinguish between different nitrogen forms^34^. The Kjeldahl method is effective for measuring total nitrogen content in plants, including both organic and ammonium forms. However, it does not distinguish between different forms of nitrogen (e.g., nitrate, ammonium, or organic nitrogen)^35^. If the plant has taken up more nitrogen due to higher concentrations in the soil, this increase in nitrogen content can be detected using the Kjeldahl method. The results of our experiment indicate that nitrogen and sulfur application significantly influence the nitrogen content in *Solanum lycopersicum*. The control group (T3) provides a useful benchmark, while T5 demonstrates the potential for maximizing nitrogen percentage through high nitrogen and sulfur application **(**Fig. 3a**).**

Nitrogen is a critical component of chlorophyll molecules. It forms the central part of the chlorophyll structure (the porphyrin ring), and its availability directly influences chlorophyll synthesis. Chlorophyll-a and chlorophyll-b both increased nitrogen availability, generally enhancing the synthesis of both chlorophyll-a and chlorophyll-b. This is because nitrogen is a key nutrient for the enzymes involved in chlorophyll biosynthesis^35^. Higher nitrogen levels typically lead to an increase in total chlorophyll content due to improved photosynthetic efficiency and plant growth. Nitrogen can also influence carotenoid content, but the effect is less direct. Carotenoids are accessory pigments that protect chlorophyll from photo-oxidation. While nitrogen primarily boosts chlorophyll, it can indirectly support carotenoid synthesis by improving overall plant health. Deficiency of nitrogen leads to chlorosis (yellowing of leaves) due to reduced chlorophyll synthesis. Decreases chlorophyll a, chlorophyll b, and total chlorophyll content. It may also reduce carotenoid content, as carotenoids are synthesized alongside chlorophyll in chloroplasts. Excess nitrogen can lead to imbalanced nutrient uptake, potentially causing toxicity or reduced uptake of other nutrients (e.g., potassium or magnesium). May not directly reduce chlorophyll content but can alter plant metabolism, indirectly affecting pigment synthesis^36^. Sulfur is a component of amino acids like cysteine and methionine, which are essential for protein synthesis, including enzymes involved in chlorophyll and carotenoid biosynthesis. A deficiency of sulfur causes chlorosis, similar to nitrogen deficiency, but often affects younger leaves first. Reduces chlorophyll a, chlorophyll b, and total chlorophyll content due to impaired protein synthesis. May also decrease carotenoid content, as sulfur is involved in the synthesis of precursors for these pigments^24,37^, excess sulfur can lead to toxicity, disrupting nutrient balance and potentially reducing chlorophyll and carotenoid content. May cause oxidative stress, leading to pigment degradation^38^. Previous studies have shown that N and S are required for chlorophyll and carotenoid synthesis^39,40^. Deficiencies or excesses of these nutrients can disrupt pigment production, leading to reduced photosynthetic efficiency and plant growth. Proper nutrient management is crucial to maintain optimal pigment levels and plant health. Chlorophyll-a increased with treatments. In the case of chlorophyll-b and total chlorophyll, T2 provided the highest amount. The carotenoid amount increased with N, S concentration (Fig. 3e). Treating plants with either a lack or excess of nitrogen and sulfur can significantly impact plant protein content and amino acid concentration. Nitrogen is a fundamental component of amino acids, which are the building blocks of proteins. A nitrogen deficiency typically leads to reduced protein synthesis, resulting in lower plant protein content and impaired growth^41^. Conversely, an excess of nitrogen can enhance protein synthesis up to a point, but beyond optimal levels, it may lead to imbalances in nutrient uptake, potentially causing toxicity or reduced efficiency in protein production. Sulfur, on the other hand, is crucial for the synthesis of sulfur-containing amino acids like cysteine and methionine, which are essential for protein structure and function^42^. A lack of sulfur can limit the production of these amino acids, thereby reducing overall protein quality and quantity. Excess sulfur, while less commonly problematic, can interfere with the uptake of other nutrients, indirectly affecting protein synthesis^27^. Studies have shown that the interplay between nitrogen and sulfur is critical; for instance, optimal N:S ratios are necessary for maximizing protein content and amino acid profiles in crops like wheat and legumes^25,43^. Therefore, both deficiencies and excesses of these nutrients can disrupt plant protein (%) and amino acid concentrations, highlighting the importance of balanced nutrient management for optimal plant health and productivity^44^.

The biochemical analysis revealed a strong correlation between N and S availability and plant metabolic responses. Protein content and amino acid levels were highest in T3, confirming efficient nitrogen assimilation. Additionally, amino acid concentration and protein percentage increased with N and S concentrations (Fig. 4). Both nitrogen and sulfur play crucial roles in regulating total soluble sugar content in plants. Imbalances in their supply (either deficiency or excess) can disrupt carbohydrate metabolism, leading to changes in TSS. Proper nutrient management is essential to maintain optimal sugar accumulation and overall plant health. Altered availability of N and S significantly affects total soluble sugar (TSS) content, as N is directly linked to photosynthesis, protein synthesis, and plant metabolism.

A nitrogen deficiency often leads to reduced chlorophyll production, impaired photosynthesis, and lower carbohydrate accumulation, thereby decreasing TSS. Conversely, excessive nitrogen can promote vegetative growth at the expense of carbohydrate storage, potentially altering sugar metabolism and reducing TSS in certain plant tissues. Sulfur, on the other hand, is essential for the synthesis of amino acids like cysteine and methionine, as well as coenzymes and vitamins. Sulfur deficiency can disrupt protein synthesis and photosynthetic efficiency, leading to reduced TSS. Excess sulfur, while less studied, may cause toxicity, impairing metabolic processes and potentially affecting sugar accumulation. Studies have shown that balanced nitrogen and sulfur fertilization optimizes TSS by supporting efficient photosynthesis and metabolic functions, whereas imbalances disrupt these processes. For example, research^45^ highlighted that optimal N and S levels enhance sugar accumulation, while deficiencies or excesses lead to significant reductions in TSS. Similarly ^46^, that sulfur deficiency specifically alters carbohydrate partitioning, reducing soluble sugar content. Thus, maintaining an appropriate balance of nitrogen and sulfur is critical for optimal TSS levels in plants.

T5 (N_500_S_500_) exhibited growth suppression, suggesting nutrient toxicity. Excessive nitrogen has been linked to ammonium toxicity, leading to metabolic imbalances^47^. Similarly, high sulfur concentrations can lead to excess sulfate accumulation, disrupting cellular homeostasis^48^. The observed increase in antioxidant enzyme activities (CAT, APX) in T5 indicates oxidative stress responses, a common defense mechanism under nutrient imbalance conditions^49^. Antioxidant enzyme activity followed a similar pattern, increasing in nutrient-deficient and excess conditions. The heightened CAT and APX activities in T5 indicate a protective response against oxidative stress induced by excessive N and S supply (Fig. 4d & e). These findings align with previous studies showing that nutrient stress triggers antioxidant defense mechanisms^50^. Treating plants with either a lack or excess of nitrogen (N) and sulfur (S) can significantly impact total cell death, as both nutrients play critical roles in plant metabolism, growth, and stress responses. Nitrogen is a key component of amino acids, proteins, and chlorophyll, while sulfur is essential for the synthesis of cysteine, methionine, and various coenzymes and antioxidants like glutathione. A deficiency in either nutrient can lead to impaired cellular functions, reduced photosynthetic efficiency, and increased oxidative stress, ultimately triggering programmed cell death (PCD) as a stress response. For example, nitrogen deficiency often results in chlorosis and senescence, while sulfur deficiency can disrupt redox balance, leading to the accumulation of reactive oxygen species (ROS) and cell death.

On the other hand, excessive nitrogen or sulfur can also be detrimental. High nitrogen levels may cause nutrient imbalances, toxicity, and excessive vegetative growth, making plants more susceptible to pathogens and environmental stresses, which can accelerate cell death. Excess sulfur, particularly in the form of sulfate, can lead to the accumulation of toxic compounds like hydrogen sulfide (H_2_S) under anaerobic conditions, disrupting cellular respiration and inducing cell death. Studies have shown that deficiencies and excesses of these nutrients can alter the expression of genes involved in stress responses and PCD, highlighting the delicate balance required for optimal plant health. For instance, sulfur helps in mitigating oxidative stress^25^, while nitrogen’s role in senescence and cell death regulation^51^. Thus, maintaining an optimal balance of nitrogen and sulfur is crucial to prevent disruptions in cellular homeostasis and avoid excessive cell death. A fluctuation was found in the cell death analysis. Lack of and excess nutrient supply both enhanced cell death. However, T5 showed the highest rate of cell death, and T3 (control) showed the lowest result in both *Solanum lycopersicum.* (Fig. 4f**).** Hydroponic systems offer precise nutrient management, minimizing environmental impacts such as environmental impacts nitrate leaching and sulfate runoff^52^.

Nitrogen and sulfur are critical for plant metabolism, influencing amino acid synthesis, protein formation, and chlorophyll production. This study demonstrates that balanced N/S supplementation (T3: 100% N/S) optimizes tomato growth, supporting higher plant height, leaf number, and biomass, consistent with prior research on nutrient synergy. Deficiencies (T1: 0% N/S) led to chlorosis and reduced biomass, while excess nutrients (T5: 500% N/S) caused toxicity, evidenced by suppressed growth and elevated antioxidant enzyme activities (CAT, APX). Limitations include the use of a single tomato cultivar and a controlled hydroponic setup with a relatively small sample size (n = 3 plants per treatment), which may limit generalizability to other cultivars or field conditions. Future studies should explore diverse cultivars and soil-based systems. The results emphasize the importance of maintaining balanced N and S levels to maximize plant productivity while preventing waste. Future studies should focus on refining nutrient formulations to enhance nutrient use efficiency in hydroponic agriculture.

## Conclusion

This study suggests that balanced nitrogen (N) and sulfur (S) supplementation (100% N/S) enhances *Solanum lycopersicum* growth, plant height, root development, leaf expansion, and overall biomass, and also increases biochemical composition and stress tolerance in hydroponic systems. The optimal nutrient ratio (T3) promoted superior morphological traits and metabolic efficiency, while deficiencies (T1) and excesses (T5) led to stress and reduced growth. Adequate N and S levels improve chlorophyll content, protein synthesis, and nitrogen uptake, which correlate with enhanced metabolic activity. Additionally, optimal nutrient levels supported increased activity of antioxidant enzymes (CAT, APX), strengthening plant defense against oxidative stress. Findings highlight the potential of precise nutrient management for sustainable hydroponic tomato production, though results are limited to a single cultivar and controlled conditions.

## Data Availability

Data will be made available on request. The datasets used and/or analyzed during the current study are available from the corresponding author upon reasonable request.
